# Real time control data for styrene acrylonitrile copolymerization system in a batch reactor for the optimization of molecular weight

**DOI:** 10.1016/j.dib.2019.104878

**Published:** 2019-11-24

**Authors:** Sharanya Suraboyina, Saipushpita Vudata, Anand Polumati

**Affiliations:** Process Engineering & Technology Transfer, CSIR- Indian Institute of Chemical Technology, Hyderabad, 500 007, India

**Keywords:** Real time control, Batch reactor, Co-polymerization, Process optimization, Molecular weight

## Abstract

This work describes an approach towards experimental implementation of real time control studies conducted on a batch polymerization reactor. The information is related to the controlling of molecular weight for styrene acrylonitrile copolymer polymerization system in a batch reactor generated under a varied range of temperatures, reactant concentrations and retention times. The operating conditions of 6 hrs and temperature of 343 K for yielding a molecular weight in the range of 39,900–40,000 gmol^−1^ is established using simulation studies. A real time control facility consisting of a batch reactor, data acquisition software “LabVIEW” and a PC for monitoring and control is used to implement these operating conditions. The resulting product is analyzed by gel permeation chromatography (GPC). The data generated can be used by researchers, academicians and industry for generating control strategies, automation and scale up of polymerization reactors.

**Table:** Specifications Table

Subject	Chemical Engineering
Specific subject area	Polymerization, Data monitoring, Control theory
Type of data	Tables, Graphs, Figures
How data were acquired	Experiments were conducted for styrene acrylonitrile polymerization process in a batch reactor. The experiment was carried out for a constant set point of 343 K (70 °C). The total experiment was carried out for six hours. The experimental data was monitored and logged using a data acquisition (DAQ) software “LabVIEW”. This is a commercial software package installed in the personnel computer to acquire, monitor, handle and analyze the data. All the control logics and the programs for data logging were written using functions in the block diagram. All the controls and indicators are displayed in the front panel to have online monitoring of the process in the form of charts during reaction time.
Data format	Raw and analyzed
Parameters for data collection	Reactor temperature, Jacket temperature, coolant flow rate, molecular weight.
Description of data collection	Experiments were conducted for styrene acrylonitrile co-polymerization process in a bench scale reactor under wide range of temperatures, reactant concentrations and retention times. A constant set point temperature in the range of 323–343K is maintained. At the end of the reaction, the sample is analyzed for GPC analyses to determine the molecular weight. A molecular weight of 40,000 is observed for the operating conditions of reaction time for 6hrs and temperature of 343 K. The experiment with optimal operating conditions is repeated in a 2 L batch reactor having a PC for online monitoring with data acquisition software. The product obtained at the end of reaction is given to GPC analysis for molecular weight determination.
Data source location	The experimental data collected at Indian Institute of Chemical Technology at Hyderabad, India.
Data accessibility	Data is with this article.
Related research article	[1] P.Anand, Ch. Venkateswarlu and M. BhagavanthaRao, “Multistage dynamic optimization of a copolymerization reactor using differential evolution”, Asia-Pac. J. Chem.Eng. 8:687–698, 2013

**Table:** 

**Value of the Data** • The monitored data can be used by researchers, academicians and industry people for generating control strategies, automation and scale up of polymerization reactors. • The dataset covering wide range experimental conditions might be used as a benchmark to validate the findings of other studies. • Data standardization provide the opportunity to compare the data acquired under different experimental conditions and highlight the importance of selecting the optimum ranges of conditions.

## 1 Data

**Fig. 1:**
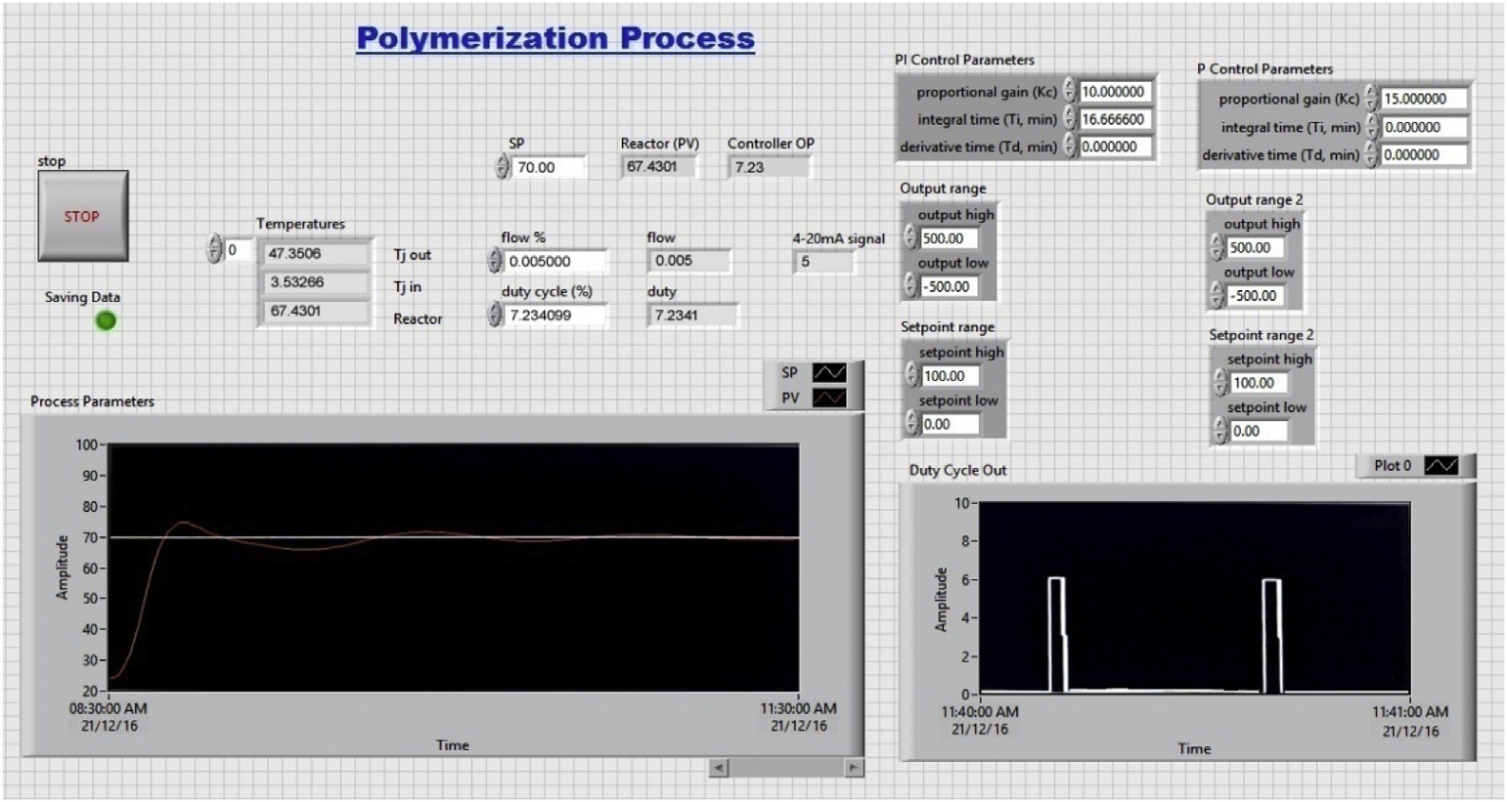
Display of temperature of the reactor and set point during the reaction.

**Fig. 2:**
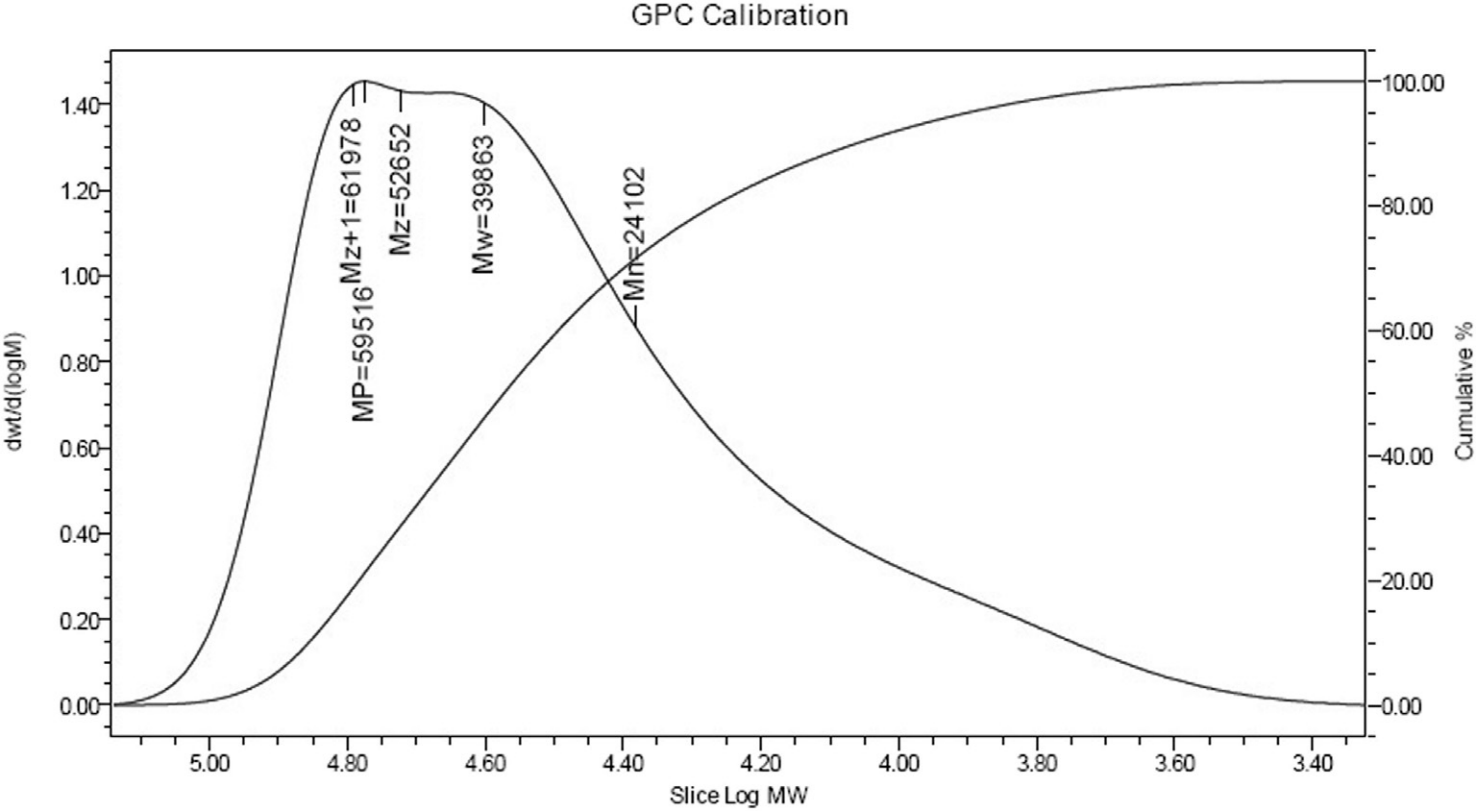
Results of GPC calibration for the obtained product.

**Fig. 3:**
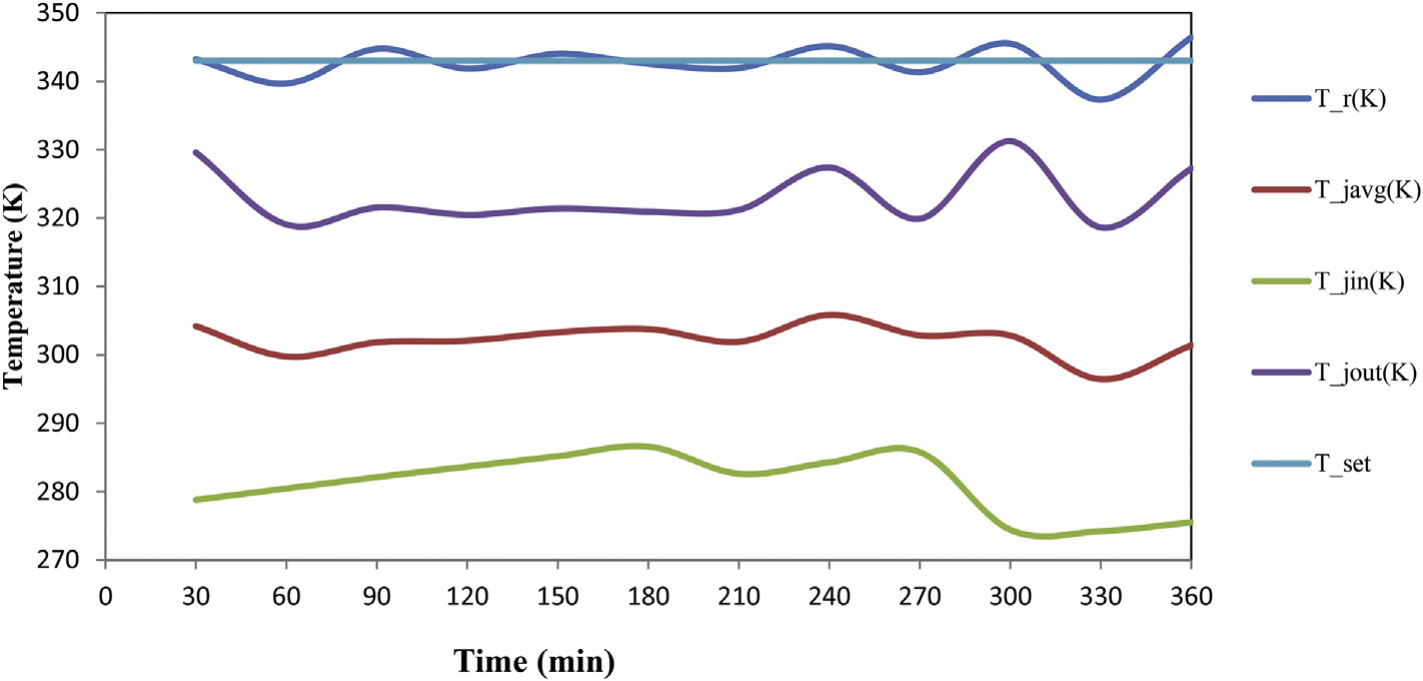
Display of temperature of the reactor and set point during the reaction.

**Fig. 4:**
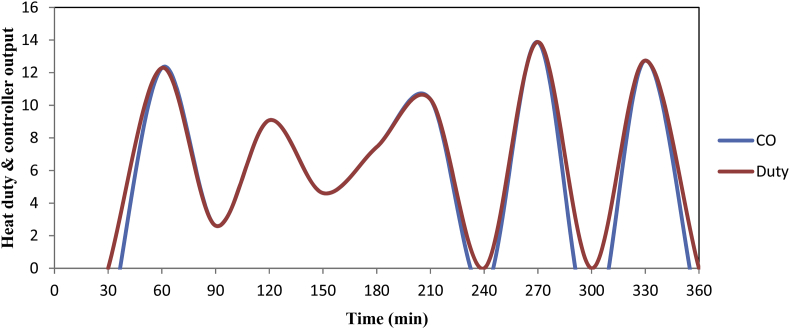
Heat duty and control action during the reaction.

**Fig. 5:**
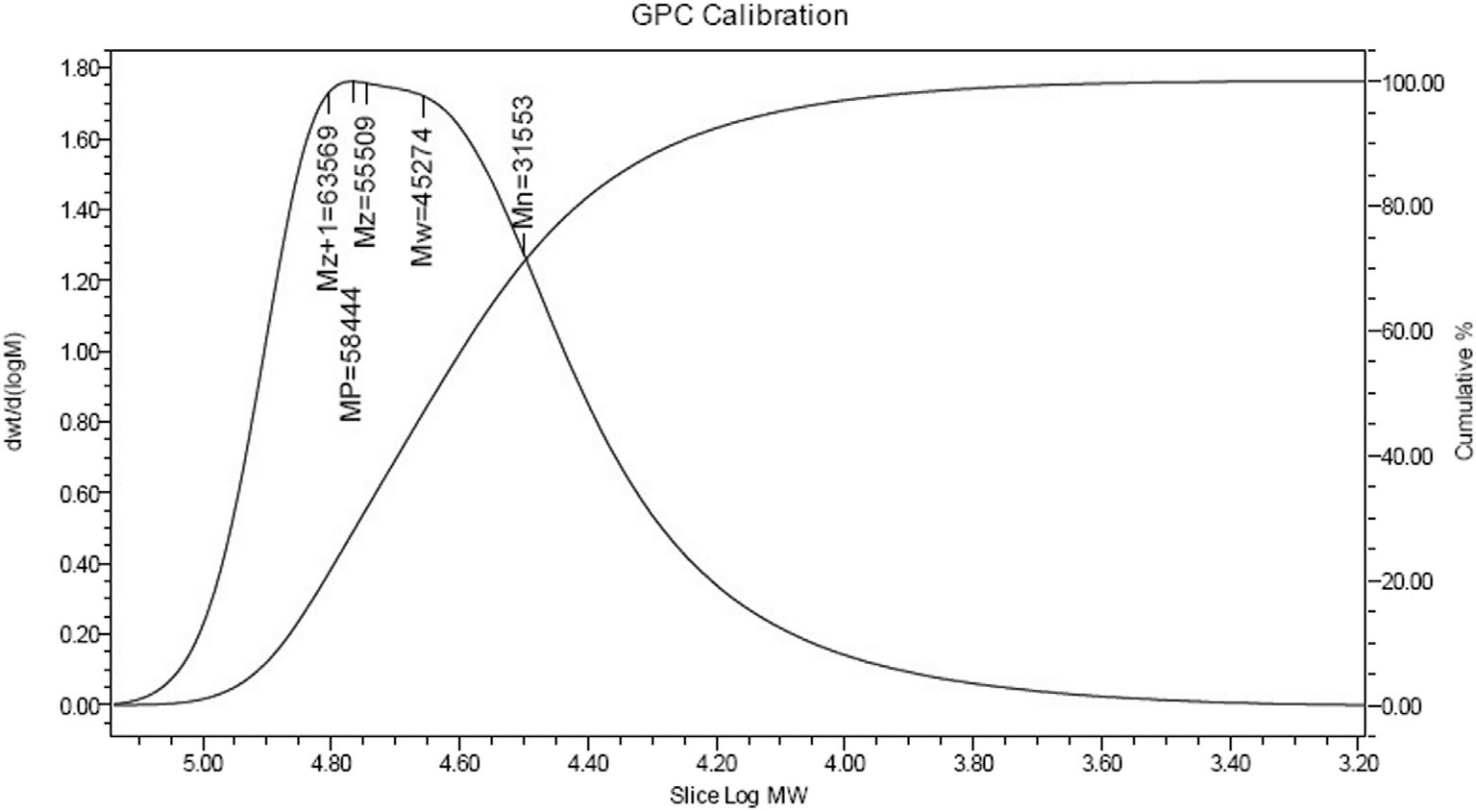
GPC analysis report for real time control.

The dataset in this article describes the experimental studies for styrene acrylonitrile copolymer polymerization in a batch reactor with varying temperatures. Real time temperature monitoring using DAQ with desired setpoint is shown in Fig. 1. Experimental conditions with varying temperatures shown in Table 1. GPC analysis results were shown in Table 2. Table 3 describes data acquired during the course of the reaction using DAQ system. Results of GPC calibration at bench scale experiment for optimum molecular weight is shown in Fig. 2. Fig. 3 shows the comparison of temperatures of reactor and jacket. Fig. 4 shows the heat duty and control action during the reaction. Fig. 5. GPC analysis report for the polymer in pilot scale experimentation.

**Table 1:** Experimental condition with varying temperatures.

Exp	Temp (K)	Styrene(g)	Acrylonitrile (g)	AIBN (g)	Xylene (g)	Polymer formed (g)	Mol. weight
1	323	90.9	81.0	0.1	86.1	0.2989	64,200
2	333	90.9	81.0	0.1	86.1	3.5138	45,300
3	343	90.9	81.0	0.1	86.1	11.576	39,900

**Table 2:** GPC analysis.

S.no	Elution volume (ml)	Retension time (min)	Mn	Mw	MP	Mz	Mz+1	Mz/Mw
1	16.70	16.72	50,880	64,140	73,800	73,720	81,030	1.15
2	17.10	17.10	31,550	45,270	58,440	55,510	63,570	1.22
3	17.07	17.07	24,100	39,990	59,520	52,650	61,980	1.32

**Table 3:** Data acquired during the course of the reaction using DAQ system.

Time (min)	Reactor Temperature(K)	Jacket inlet temperature(K)	Jacket outlet temperature (K)	Average temperature (K)	set point (K)	Control output	Duty cycle	Coolant Flow
30	343.2373	278.7965	329.610	304.2036	343	−4.1605	0.0000	0.0056
60	339.6759	280.4680	319.048	299.7580	343	12.2869	12.2869	0.0050
90	344.7579	282.1240	321.5317	301.8279	343	2.6171	2.6171	0.0050
120	341.8712	283.6707	320.4821	302.0764	343	9.0822	9.0822	0.0050
150	344.0171	285.1796	321.4157	303.2976	343	4.6150	4.6150	0.0050
180	342.5700	286.5934	320.9544	303.7739	343	7.4371	7.4371	0.0050
210	341.9505	282.6208	321.1993	301.9100	343	10.3653	10.3653	0.0050
240	345.1563	284.2869	327.4002	305.8435	343	−1.4093	0.0000	0.0052
270	341.3274	285.8014	319.9136	302.8575	343	13.8799	13.8799	0.0050
300	345.5229	274.4257	331.2528	302.8392	343	−3.9297	0.0000	0.0055
330	337.3121	274.2209	318.6480	296.4344	343	12.7421	12.7421	0.0050
360	346.4253	275.5112	327.2502	301.38,078	343	−3.1347	0.0000	0.0054

## 2 Process and model description

In this work, styrene acrylonitrile copolymer is chosen where it is also modeled prior to experimentation [2,3]. The feed is a mixture of monomers (*M*_1_, *M*_2_), solvent and initiator (*I*). The mathematical model of the process is given as follows:

(1) $$dM_{1}/dt=u\left(M_{1f}-M_{1}\right)/V-\left[\left(k_{p11}+k_{f11}\right)P+\left(k_{p21}+k_{f21}\right)Q\right]M_{1}$$

(2) $$dM_{2}/dt=u\left(M_{2f}-M_{2}\right)/V-\left[\left(k_{p22}+k_{f22}\right)Q+\left(k_{p12}+k_{f12}\right)P\right]M_{2}$$

(3) $$dI/dt=u\left(I_{f}-I\right)/V-k_{d}I$$

(4) dV/dt=u

The moment equations for dead polymers are given by three moments:

(5) $$d\lambda_{0}^{d}/dt=\left(k_{tc11}/2+k_{td11}\right)P^{2}+\left(k_{tc22}/2+k_{td22}\right)Q^{2}+\left(k_{tc12}+2k_{td12}\right)PQ+\left(k_{f11}M_{1}+k_{f12}M_{2}\right)P+\left(k_{f22}M_{2}+k_{f21}M_{1}\right)Q-\left(\lambda_{0}^{d}/V\right)u$$

(6) $$d\lambda_{1}^{d}/dt=\left(k_{tc11}P+k_{td11}P+k_{tc12}Q+k_{td12}Q+k_{f11}M_{1}+k_{f12}M_{2}\right)P_{1}+\left(k_{tc22}Q+k_{td22}Q+k_{tc12}P+k_{td12}P+k_{f22}M_{2}+k_{f12}M_{1}\right)Q_{1}-\left(\lambda_{1}^{d}u\right)/V$$

(7) $$d\lambda_{2}^{d}/dt=\left(kt_{c11}P+k_{td11}P+k_{tc12}Q+k_{td12}Q+k_{f11}M_{1}+k_{f12}M_{2}\right)P_{2}+\left(k_{tc22}Q+kt_{d22}Q+k_{tc12P}+k_{f22}M_{2}+k_{f21}M_{1}\right)Q_{2}+k_{tc11}P_{1}^{2}+k_{t_{c22}}Q_{1}^{2}+2k_{tc12}P_{1}Q_{1}-\lambda_{2}^{d}u/V$$

The rate constants, $k_{d}$, $k_{fij}$, $k_{pij}$, $k_{tcij}$ and $k_{tdij}$ are Arrhenius functions of temperature, the pre-exponential factors and activation energies of which are reported [4].

## 3 Experimental design, materials, and methods

Experiments were conducted for styrene acrylonitrile polymerization process in a batch reactor with the conditions mentioned in Table 1. Styrene and acrylonitrile of technical grade were used as the reactants. The solvent used is xylene and initiator is AIBN. The experiment was carried out for a constant set point of 343 K (70 °C). The total experiment was carried out for six hours. The product is just cooled to de-activate the polymerization and then precipitated the product into methanol to isolate the polymer. The sample at the end of reaction is given to GPC analysis for molecular weight determination. The monomer used in this study was obtained with 99% purity from Sigma Aldrich. The feedstock was maintained under a temperature of 4 °C in a cold room. The solvent used was xylene purchased from Aldrich with a purity of 99%, and was used without any further purification. Initially, the mixture of styrene and acrylonitrile was heated up to the desired initial temperature of 70 °C.

## 4 Data aquisition software

The data was monitored using a data aquisition software “LabVIEW” which is a commercial software package installed in the personnel computer to acquire, monitor, handle, analyze and log the data. All the control logic and the program for data logging is written using functions in the block diagram. All the controls and indicators are displayed in the front panel where we can also have online monitoring of the process in the form of charts at runtime. In the block diagram the process parameters are read using the input DAQ Assistant function. These values are logged into text files using the write to measurement file function. The process variable is given to the PI control function and the obtained controller output is fed back to the O/P modules using the output DAQ Assistant function. The required process values are given to a chart to have an online trend while running the process which can be seen on the front panel.

## 5 Bench scale experiments

Free radical polymerization experiments were carried out in 250 ml glass reactor to establish and optimize the process conditions. The experiment is conducted with varying temperatures (323, 333 and 343K) is listed in Table 1. The molecular weight is analyzed through GPC analysis and a molecular weight of approximate value of 39,900–∼40,000 is observed. GPC analysis as tabulated in Table 2, indicate the conformity of results with the simulated results Anandet.al, 2013, 2014 [2,3].

Real time temperature monitoring using DAQ with desired set point is shown in Fig. 1. Results of GPC calibration at bench scale experiment for optimum molecular weight is shown in Fig. 2. Fig. 3 shows the comparison of temperatures of reactor and jacket. Fig. 4. Shows the heat duty and control action during the reaction. Fig. 5. GPCanalysis report for the polymer in pilot scale experimentation.

## 6 Gel permeation chromatography (GPC)

The polymer synthesized at the end of the reaction time is subjected to GPC analysis for determination of molecular weight. The sample is first dissolved in solvent (THF-tetra hydro furan) and is injected into a continually flowing stream of tetrahydrofuran (THF) without disturbing the continuous mobile phase flow at a flow rate of 0.05 ml/min. The mobile phase flows through millions of highly porous, rigid particles tightly packed together in a column. The column **separates sample components from one another with** rapid analyses. Detector used is refractive index to monitor molecular weight distribution which is directly proportional to concentration. In this procedure the molecular weight of the sample is determined based on the standards obtained by plotting the retention time against the log of molecular weight. The operating conditions for these methods are established as flow rate of 0.05 ml/min, detectors were refractive index, the temperature is 24 °C and the column porosities are10000 Å. The molecular weight of SAN copolymer obtained through this method is 30,000.

## 7 Pilot scale experiments

The operating conditions of reaction time for 6 hrs and temperature of 343 K yield a molecular weight of 39,900 g mol-1 which is nearer to the desired molecular weight of 40,000 g mol-1 is selected as the best operating conditions. The experiment with optimal operating conditions is repeated in a 2 L batch reactor having a PC for online monitoring with data acquisition software. The experimental setup consists of a stainless-steel reactor (Parr) of 2 L capacity with an external jacket with an electric coil for heating, and also with an internal cooling coil. A total volume of 1200 ml of mixture (70% of styrene and 30% of acrylonitrile) was used to carry out the experiments. The experiments were carried out by monitoring and controlling the reactor temperature using the manipulated heater with the aid PID controller algorithms accordingly. The optimum operating conditions that are used in pilot scale experiments were temperature: 343K, styrene: 160ml, acrylonitrile:160ml,xylene:800ml and azobisisobutyronitrile: 1.6 g.
